# Perforation risk factors in ESR-EB resection of gastric submucosal tumors ≤ 10 mm

**DOI:** 10.1097/MD.0000000000049847

**Published:** 2026-07-17

**Authors:** Zhaohui Liu, Wenjun Zhou, Peng Wu, Dayong Sun, Ruinuan Wu

**Affiliations:** a The Department of Gastroenterology, Shenzhen Second People’s Hospital, First Affiliated Hospital of Shenzhen University Health Science Center, Shenzhen, Guangdong, China; b Department of Medicine, Shenzhen University, Shenzhen, Guangdong, China; c Department of Medicine, Shantou University, Shenzhen, Guangdong, China; d The Department of Pathology, Shenzhen Second People’s Hospital, First Affiliated Hospital of Shenzhen University Health Science Center, Shenzhen, Guangdong, China.

**Keywords:** ESR-EB, GISTs, perforation, risk factors

## Abstract

This study aimed to investigate the risk factors for perforation during endoscopic snare resection with an elastic band (ESR-EB) resection of gastric submucosal tumors ≤ 10 mm, providing clinical guidance for preventing intraoperative adverse events. A retrospective study was conducted on patients who underwent ESR-EB resection of gastric submucosal tumors ≤ 10 mm at Shenzhen Second People’s Hospital from April 2023 to October 2024. Clinical characteristics, tumor location, size, pathological type, resection time, operation time, and perforation rate were analyzed using binary regression analysis to identify risk factors for perforation. A total of 108 patients underwent ESR-EB surgery, with a mean age of 55.83 ± 9.27 years and 30.56% being male. The mean tumor size was 6.07 ± 1.42 mm, with intraluminal growth being the most common (88.89%). Tumors were primarily located in the fundus (72.22%) and body (27.78%) of the stomach. The mean operation and resection times were 21.22 ± 10.37 minutes and 9.02 ± 4.99 minutes, respectively. Gastrointestinal stromal tumors (GISTs) were the most common pathological type (75.93%), followed by leiomyomas (21.30%). Perforation occurred in 73.15% of cases during surgery, all of which were successfully closed endoscopically. Binary regression analysis showed that tumor location and histological type were risk factors for perforation. Tumors in the fundus increased perforation risk (odds ratio = 5.080, 95% confidence interval 1.234–20.923; *P* = .024). The risk of perforation was significantly reduced in non-GIST pathological types (odds ratio = 0.129,95% confidence interval 0.029–0.565; *P* = .007). Gender, age, tumor size, growth pattern, operation time, and resection time were not associated with perforation. ESR-EB can completely resect gastric submucosal tumors ≤ 10 mm. Perforation risk is higher when tumors are located in the fundus or are GISTs. Tumor location is a useful preoperative predictor of intraoperative perforation.

## 1. Introduction

For gastric submucosal tumors ≤ 10 mm, endoscopic resection is necessary. Previous studies^[1–4]^ have shown that even small gastrointestinal stromal tumors (GISTs) ≤ 10 mm have the potential for malignancy and can grow during follow-up.^[5]^ Tanaka J et al^[6]^ reported a case of a 10 mm submucosal tumor near the cardia that grew to 3 cm within 3 months, forming an ulcer and being diagnosed as high-risk GIST postoperatively, which later metastasized to the liver and caused death. These reports indicate that small GISTs ≤ 10 mm still pose a risk and require early intervention.

Several endoscopic techniques are available for resecting gastric submucosal tumors. Endoscopic snare resection with an elastic band (ESR-EB) is a simple, inexpensive method that combines variceal-ligation hardware with standard polypectomy: the lesion is suctioned into a transparent cap, strangulated with an elastic band to produce avascular pseudo-stalk, and subsequently transected with a diathermy snare placed distal to the band, allowing full-thickness en-bloc removal of tumors ≤ 10 mm.Which has been found to be an effective and time-saving technique in recent studies.^[7–10]^ However, a high perforation rate during surgery is a concern with this technique. No studies have yet reported on the correlation between ESR-EB and intraoperative perforation. This study retrospectively analyzed the risk factors for intraoperative perforation during ESR-EB to provide guidance for its prevention and treatment.

## 2. Materials and methods

### 2.1. Study design and ethics

This was a single-center retrospective study conducted at Shenzhen Second People’s Hospital. The study was conducted in accordance with the revised 2008 Helsinki Declaration and was approved by the hospital’s ethics committee (approval No.2026-007-01PJ). Data were collected from patients who underwent ESR-EB resection of gastric submucosal tumors from April 2023 to October 2024. Informed consent for endoscopic resection was obtained from each patient. The study did not require individual consent due to its retrospective nature.

### 2.2. Indications for ESR-EB

The indications for ESR-EB resection included: tumors originating from the gastric muscularis propria; tumors with a size ≤ 10 mm on endoscopic ultrasound; no lymph node or distant metastasis on preoperative computed tomography; stable general condition without severe cardiopulmonary insufficiency, allowing for endoscopic surgery. The surgical approach was chosen based on the operator’s personal preference.

### 2.3. Surgical instruments and procedure

The following instruments were used: an endoscopic image processor (CLV-290sl, Olympus), a therapeutic gastroscope (HQ260J, Olympus), a high-frequency electrosurgical generator (VIO300D, Erbe), a snare (M00561231, Boston Scientific), tissue clips (POCC-D-26-195, MicroPort), and an endoscopic ligation device (M00542251, Boston Scientific).

The surgical procedure involved attaching a ligator to the front end of the endoscope. The endoscope was inserted into the stomach to locate the lesion. The transparent cap was used to suction the lesion into it, and an elastic band was released to ligate it. The snare was placed below the elastic band, connected to the electrosurgical generator, and used to perform slow electrocautery resection. The resection site was inspected for perforation or bleeding. If bleeding occurred, the vessel stump was electrocoagulated with hemostatic forceps. The defect was closed with tissue clips. The resected lesion was removed from the body, its completeness was observed, a photo was taken, and its size was measured. The specimen was then placed in 10% formalin for pathological examination.

### 2.4. Clinical outcomes

The following outcomes were analyzed: complete endoscopic resection rate, operation time, resection time, intraoperative perforation, postoperative hospital stay, and pathological results. Complete endoscopic resection was defined as the removal of the tumor with no visible residual mass and negative pathological margins. Operation time was defined as the interval from the insertion of the endoscope into the mouth to its removal. Resection time was defined as the interval from the suction of the lesion into the transparent cap to the closure of the defect with tissue clips. Perforation was defined as the direct visualization of abdominal organs or omentum. Postoperative hospital stay was defined as the interval from the completion of surgery to discharge.

### 2.5. Statistical analysis

All data were analyzed using SPSS 28 (IBM Corp., Armonk). Count data (e.g., gender, growth pattern, lesion location, and pathological results) were described using frequencies. Continuous variables (e.g., age, tumor size, operation time, resection time, and postoperative hospital stay) were expressed as mean ± standard deviation. All candidate variables were first examined in univariate logistic regressions. Covariates with *P* > .10 were nevertheless retained in the initial multivariable model when clinically plausible. A backward-elimination procedure (removal criterion *P* > .10) was then applied to confirm the stability of the final estimates (Table 1).Binary regression analysis was used to assess the relationship between clinical factors and treatment outcomes. All reported *P* values were 2-tailed, and *P* < .05 was considered statistically significant.

**Table 1:** Univariate analysis for perforation (n = 109).

Variable	OR (95% CI)	*P* value
Age (per 1 year)	1.02 (0.97–1.07)	.42
Size (per 1 cm)	0.74 (0.55–0.99)	.046
Growth pattern	0.50 (0.15–1.63)	.25
Location	1.07 (0.48–2.38)	.87
Operation time (per 1 min)	1.00 (0.98–1.03)	.7
Resection time (per 1 min)	1.00 (0.96–1.04)	.9
Pathology	2.20 (0.86–5.64)	.1

CI = confidence interval, n = number of patients, OR = odds ratio.

## 3. Results

### 3.1. Patient characteristics and treatment outcomes

A total of 108 patients underwent ESR-EB surgery. The mean age was 55.83 ± 9.27 years, with 30.56% being male. The mean tumor size was 6.07 ± 1.42 mm, with intraluminal growth being the most common (88.89%). Tumors were primarily located in the fundus (72.22%) and body (27.78%) of the stomach. The mean operation and resection times were 21.22 ± 10.37 minutes and 9.02 ± 4.99 minutes, respectively. GISTs were the most common pathological type (75.93%), followed by leiomyomas (21.30%). Perforation occurred in 73.15% of cases during surgery, all of which were successfully closed endoscopically (Table 2).

**Table 2:** Patient characteristics and treatment outcomes.

Outcomes	Mean ± SD (n, %)
n	108
Age (yr)	55.83 ± 9.27
Gender (male)	33 (30.56%)
Size of tumor (mm)	6.07 ± 1.42
Location of tumor	
Body	30 (27.78)
Fundus	78 (72.22)
Operation time (min)	21.22 ± 10.37
Resection time (min)	9.02 ± 4.99
Growth pattern,	
Intraluminal growth	96 (88.89)
Extraluminal growth	2 (1.85)
Mixed growth	10 (9.26)
Histology diagnosis	
Leiomyoma	23 (21.30)
GIST	82 (75.93)
Others	3 (2.78)
Perforation	79 (73.15)

GIST = gastrointestinal stromal tumor, n = number of patients, SD = standard deviation.

### 3.2. Binary regression analysis for perforation

Perforation occurred in 79 patients. Binary regression analysis showed that tumor location and histological type were risk factors for perforation. Tumors in the fundus increased perforation risk (odds ratio [OR] = 5.080, 95% confidence interval 1.234–20.923; *P* = .024). The risk of perforation was significantly reduced in non-GIST pathological types (OR = 0.129, 95% confidence interval 0.029–0.565; *P* = .007) (Table 3). Gender, age, tumor size, growth pattern, operation time, and resection time were not associated with perforation (Table 4).

**Table 3:** Robustness of “tumor location” after correcting for overrepresentation of fundus lesions.

Analysis	Fundus prevalence forced to (%)	OR for perforation (fundus vs body)	95 % CI	*P* value
Original cohort	72	5.08	1.23–20.92	.024*
Propensity-matched (1:1, n = 58)	50	4.8	1.2–19.3	.028*
Bootstrap resampling (1000 runs)	50	4.9 (median)	1.3–19.1	-

CI = confidence interval, n = number of patients, OR = odds ratio.

* *P* < .05.

**Table 4:** Binary regression analysis for perforation.

Characteristics	RC	SE	*z* value	Wald *χ*^2^	*P* value	OR value	95% CI
Gender	−0.258	0.660	−0.391	0.153	.696	0.773	0.212–2.815
Age	0.060	0.037	1.630	2.657	0.103	1.062	0.988–1.142
Size of tumor	−0.365	0.233	−1.572	2.470	.116	0.694	0.440–1.095
Growth pattern	0.470	0.931	0.505	0.255	.613	1.601	0.258–9.929
Location of tumor	1.625	0.722	2.251	5.065	.024*	5.080	1.234–20.923
Operation time	0.092	0.058	1.573	2.475	.116	1.096	0.978–1.229
Resection time	−0.039	0.113	−0.342	0.117	.732	0.962	0.771–1.200
Histology diagnosis	−2.050	0.755	−2.716	7.376	.007*	0.129	0.029–0.565

CI = confidence interval, OR = odds ratio, RC = regression coefficient, SE = standard error.

* *P* < .05.

### 3.3. Clinical impact of perforation

Comparison between the perforated group and the non-perforated group: hospital stay: median 6.0 days vs 6.0 days, Mann–Whitney *U* = 1218, *P* = .92. Antibiotic use: 100 % (79/79) vs 44.83 % (13/29), *χ*^2^ = 36.4, *P* = .001; all antibiotics were single-dose prophylaxis with no extra cost. No bleeding, surgery or readmission occurred (Table 5).

**Table 5:** Clinical outcomes of patients with vs without intraoperative perforation (n = 108).

Outcome	Perforation (+) n = 79	Perforation (–) n = 29	*P* value
Postoperative hospital stay (d), median (IQR)	6.0 (5–7)	6.0 (5–6)	.92†
Antibiotic use‡, n (%)	79 (100)	13 (44.83)	.001*
Delayed bleeding, n (%)	0 (0)	0 (0)	-
Surgical conversion, n (%)	0 (0)	0 (0)	-
Readmission within 30 d, n (%)	0 (0)	0 (0)	-

IQR = interquartile range, n = number of patients.

† Mann–Whitney *U* test.

‡ Single-dose prophylactic intravenous antibiotic only.

* *P* < .05.

## 4. Discussion

In recent years, numerous studies have reported on the use of ESR-EB for resecting gastric submucosal tumors.^[11,12]^ Limited by the inner diameter of the ligator cap, ESR-EB is suitable only for tumors ≤ 10 mm. Previous reports have shown a high complete resection rate but also a high perforation rate with ESR-EB,^[13]^ which is consistent with our findings.

Perforation is a common adverse event in endoscopic operations. A study reported that the widely used endoscopic submucosal excavation technique is associated with a perforation rate of 26.47% when resecting small gastric muscularis propria tumors.^[7]^ The perforation rate of endoscopic submucosal excavation is substantially lower than that of ESR-EB in our series.^[8,9]^ This occurs because the tumor and the entire stomach wall are suctioned into the transparent cap and ligated during the procedure, leading to full-thickness resection and perforation. In this study, 73.15% of the 108 patients experienced perforation, all of which were small and successfully closed endoscopically, consistent with previous reports.^[11–13]^ Despite the high perforation rate, perforation did not prolong postoperative hospitalization, likely because of routine antibiotic prophylaxis and well-established endoscopic closure techniques.

The perforation rate of 73.15% in this study appears alarmingly high compared with conventional endoscopic procedures such as endoscopic submucosal dissection (ESD) (2.8–8.2%).^[14]^ However, this high incidence must be interpreted within the context of ESR-EB’s technical mechanism. Unlike unplanned perforations in ESD, ESR-EB-induced perforations are inevitable consequences of the full-thickness resection design. The procedure intentionally draws the tumor and entire gastric wall into the transparent cap for ligation, followed by transection distal to the band, which by definition creates a controlled full-thickness defect.

Importantly, these perforations are fundamentally different from complicated perforations in other settings: they are small, localized defects (typically < 5mm in diameter) created under direct visualization; the brief resection time limits peritoneal contamination; the elastic band provides immediate mechanical compression of vessels, preventing significant bleeding; and the defects are amenable to reliable endoscopic closure with tissue clips. Our data demonstrate that despite the high perforation rate, there was no procedure-related bleeding, no need for emergency surgery, no prolonged hospitalization, and no additional medical costs beyond single-dose prophylactic antibiotics.

This “high perforation rate, low clinical impact” paradox is consistent with previous reports on ligation-assisted techniques. Liu et al^[9]^ reported a 72.41% perforation rate with ESR-C, yet all cases were managed endoscopically without sequelae. Similarly, Pan and Shi reported perforation in 10 of 42 gastric fundus lesions treated with ESR-EB, all successfully closed endoscopically.^[15]^ These findings suggest that for ESR-EB, perforation should be regarded as an expected technical outcome rather than a complication, provided that endoscopic closure capability is available.

The clinical implication is clear: ESR-EB should be performed in centers with expertise in endoscopic closure techniques, and patients should be counseled that “perforation” in this context does not carry the conventional connotations of severe adverse events. The technique trades a high but manageable perforation rate for superior efficiency and safety profile (minimal bleeding risk, short procedure time), making it particularly suitable for small gastric subepithelial tumors ≤ 10mm.

Bleeding is another concerning intraoperative adverse event. Since most patients undergo full-thickness resection, it is difficult to assess the vessels on the serosal side during surgery, and serosal bleeding may be uncontrollable. To avoid this adverse event, all procedures in our center were performed using coagulation cutting, which prevented bleeding. Therefore, no bleeding occurred in any of the patients.

No studies have yet reported on the causes of intraoperative perforation in ESR-EB. This study used binary regression analysis to show that tumors in the fundus are associated with a higher perforation rate than those in the body. This may be because the stomach wall in the fundus is thinner, making it more susceptible to full-thickness ligation during suction. In contrast, the thicker stomach wall in the body makes full-thickness ligation less likely. This finding suggests that when performing ESR-EB on tumors in the fundus, it is necessary to prepare for perforation closure in advance to reduce closure time and gastric juice leakage. To address this issue, our center has previously conducted a study on pre-closure (pre-closure refers to the preemptive placement of endoclips around the lesion before its resection, thereby reducing the tension that could lead to impending perforation), which significantly reduced surgical time and made closure easier.^[15]^

The study also found that GISTs are associated with a higher perforation rate,which may have been due to the poor tumor capsule and tight adhesions.^[16]^ During suction, the tumor may bring the serosa into the transparent cap, leading to full-thickness ligation of the stomach wall. Since it is difficult to determine the histological type of tumors ≤ 10 mm preoperatively, it is not possible to prevent intraoperative perforation based on histological type. Therefore, tumor location is a more effective factor for predicting intraoperative perforation.

The predominance of fundic lesions (72.22%) in our cohort raises important considerations regarding generalizability. While this distribution deviates from the expected anatomical prevalence of gastric GISTs, it paradoxically strengthens our primary conclusion: even within a cohort enriched for fundic lesions (where operators would presumably be most experienced with this location), fundic position remained an independent predictor of perforation (OR = 5.080). This suggests that the technical challenges of fundic ESR-EB (thinner wall, difficult angulation) persist despite operator familiarity. External validation in cohorts with more balanced anatomical distribution is warranted to confirm the generalizability of our risk factor model.

This study has several limitations. First, as a single-center retrospective study, the sample size is limited (n = 108), and the high proportion of fundic lesions (72.22%) indicates selection bias. While sensitivity analyses confirmed the robustness of our findings, the generalizability to centers with different case mixes requires validation. The predominance of fundic lesions likely reflects referral patterns and operator technique evolution during the study period. Second, 4 endoscopists performed ESR-EB, and we cannot eliminate inter-operator variability, though all had > 5 years experience and > 200 ESD procedures. Third, the lack of preoperative histological diagnosis limits the clinical utility of the “GIST vs non-GIST” risk factor, as this information is unavailable for preoperative planning.

In conclusion, ESR-EB can effectively resect gastric submucosal tumors ≤ 10 mm, despite the high intraoperative perforation rate, which can be successfully closed endoscopically. When tumors are located in the fundus, it is necessary to prepare for perforation closure in advance. Since the histological type cannot be determined preoperatively, it has no clinical value for preoperative guidance.

## Author contributions

**Data curation:** Wenjun Zhou, Peng Wu.

**Resources:** Zhaohui Liu, Wenjun Zhou.

**Software:** Peng Wu.

**Supervision:** Zhaohui Liu, Dayong Sun, Ruinuan Wu.

**Writing** – **original draft:** Zhaohui Liu.

**Writing** – **review & editing:** Ruinuan Wu.
